# Pathological histone acetylation in Parkinson’s disease: Neuroprotection and inhibition of microglial activation through SIRT 2 inhibition

**DOI:** 10.1016/j.neulet.2017.12.037

**Published:** 2018-02-14

**Authors:** Ian F. Harrison, Andrew D. Smith, David T. Dexter

**Affiliations:** a UCL Centre for Advanced Biomedical Imaging, Division of Medicine, University College London, 72 Huntley Street, London, WC1E 6DD, UK; b Parkinson’s Disease Research Group, Centre for Neuroinflammation and Neurodegeneration, Division of Brain Sciences, Department of Medicine, Imperial College London, Hammersmith Hospital Campus, London, W12 0NN, UK

**Keywords:** BCL2, B-cell lymphoma 2, BDNF, brain derived neurotrophic factor, COX2, cyclooxygenase 2, GDNF, glial derived neurotrophic factor, HAT, histone acetyltransferase, HDAC, histone deacetylase, HLA-DPα1, human leukocyte antigen DPα1, IL1β, Interleukin 1β, IL6, Interleukin 6, iNOS, inducible nitric oxide synthase, LCM, laser capture microdissection, LPS, lipopolysaccharide, NO, nitric oxide, PD, Parkinson’s disease, SIRT 2, Silent Information Regulator 2, SNpc, Substantia Nigra pars compacta, TH, tyrosine hydroxylase, TNFα, tumour necrosis factor α, αSyn, αSynuclein, Parkinson’s disease, Histone deacetylase inhibitor, SIRT 2, Neurodegeneration, Neuroprotection, Microglia

## Abstract

Parkinson’s disease (PD) is associated with degeneration of nigrostriatal neurons due to intracytoplasmic inclusions composed predominantly of a synaptic protein called α-synuclein. Accumulations of α-synuclein are thought to ‘mask’ acetylation sites on histone proteins, inhibiting the action of histone acetyltransferase (HAT) enzymes in their equilibrium with histone deacetylases (HDACs), thus deregulating the dynamic control of gene transcription. It is therefore hypothesised that the misbalance in the actions of HATs/HDACs in neurodegeneration can be rectified with the use of HDAC inhibitors, limiting the deregulation of transcription and aiding neuronal homeostasis and neuroprotection in disorders such as PD. Here we quantify histone acetylation in the Substantia Nigra pars compacta (SNpc) in the brains of control, early and late stage PD cases to determine if histone acetylation is a function of disease progression. PD development is associated with Braak-dependent increases in histone acetylation. Concurrently, we show that as expected disease progression is associated with reduced markers of dopaminergic neurons and increased markers of activated microglia. We go on to demonstrate that *in vitro,* degenerating dopaminergic neurons exhibit histone hypoacetylation whereas activated microglia exhibit histone hyperacetylation. This suggests that the disease-dependent increase in histone acetylation observed in human PD cases is likely a combination of the contributions of both degenerating dopaminergic neurons and infiltrating activated microglia. The HDAC SIRT 2 has become increasingly implicated as a novel target for mediation of neuroprotection in PD: the neuronal and microglial specific effects of its inhibition however remain unclear. We demonstrate that SIRT 2 expression in the SNpc of PD brains remains relatively unchanged from controls and that SIRT 2 inhibition, via AGK2 treatment of neuronal and microglial cultures, results in neuroprotection of dopaminergic neurons and reduced activation of microglial cells. Taken together, here we demonstrate that histone acetylation is disease-dependently altered in PD, likely due the effects of dopaminergic neurodegeneration and microglial infiltration; yet SIRT 2 remains relatively unaltered with disease. Given the stable nature of SIRT 2 expression with disease and the effects of SIRT 2 inhibitor treatment on degenerating dopaminergic neurons and activated microglia detected *in vitro*, SIRT 2 inhibitors warrant further investigation as potential therapeutics for the treatment of the PD.

## Highlights

• Parkinson’s is associated with Braak dependent histone hyperacetylation in the SNpc.
• SNpc SIRT 2 expression remains relatively stable with disease progression.
• Degenerating dopaminergic neurons in vitro exhibit histone hypoacetylation.
• Activated microglia in vitro exhibit histone hyperacetylation.
• Pharmacological SIRT 2 inhibition reduces neurodegeneration and microglial activation.

## 1 Introduction

Parkinson’s disease (PD) is the second most common neurodegenerative disease, and the most prevalent movement disorder, presenting clinically as cardinal symptoms of rigidity, tremor, and bradykinesia [11,26]. These primary motor symptoms are the result of degeneration of dopaminergic nigrostriatal pathways, thought to be due to intracytoplasmic protein inclusions in dopaminergic neurons within the Substantia Nigra pars compacta (SNpc), known as Lewy bodies and Lewy neurites, composed predominantly of a synaptic protein called α-synuclein (αSyn) [11,48]. Dopaminergic SNpc neurodegeneration in PD is also associated with activation of the brain’s innate immune response, with an activation of resident microglia in the brain [11,26]. Recruitment of activated microglia to the SNpc in PD therefore leads to an upregulation and secretion of proinflammatory cytokines, such as Tumour Necrosis Factor α (TNFα), Interleukin 6 (IL6) and Interleukin 1β (IL1β), and activation of inducible Nitric Oxide Synthase (iNOS) resulting in production of nitric oxide (NO) [23], which in turn are thought to exacerbate degeneration of dopaminergic SNpc neurons [11]. In recent years epigenetic mechanisms such as DNA methylation and histone remodelling through acetylation have also become implicated in PD pathogenesis [2], and as such have become increasingly studied in PD pathogenesis.

In healthy cells there is a tightly controlled equilibrium between the effects of histone acetyltransferases (HATs) and histone deacetylases (HDACs) enabling histone (de)acetylation and the dynamic control of gene transcription [13,45]. In healthy neurons this results in appropriate regulation of gene expression and subsequently facilitates appropriate neuronal homeostasis [45]. In neurodegenerative disease however, there is known to be an imbalance between the activities of HATs and HDACs in favour of histone deacetylation, thought to be pathogenic in disease progression [13,43,45]. This misbalance in neurodegeneration was first noted in both an *in vitro* model of cortical neuronal cell death induced by activation of amyloid precursor protein signalling, a hallmark of Alzheimer’s disease, and in an *in vivo* model of amyotrophic lateral sclerosis: the G86R mutant SOD-1 mice displaying motor neuron degeneration [43]. More specific to PD, we demonstrated recently that intracellular protein accumulation in a ubiquitin proteasome inhibitor rat model of PD results in histone hypoacetylation [21]. Likewise αSyn accumulation itself has been shown to promote histone H3 hypoacetylation as ascertained from overexpression studies in SH-SY5Y cells as well as in an *in vivo* αSyn transgenic drosophila model, thought to be achieved through αSyn ‘masking’ acetylation sites on histone proteins [31]. From these findings then it is hypothesised that the misbalance in the activities of HATs/HDACs could be rectified with the use of HDAC inhibitors (HDACIs) to reduce the extent of cell death in the nigrostriatal pathways in PD [10,13,19,22,29]. For example, inhibitions of HDACs 1 and 2 [9], and 6 [27,38], as well as broader inhibitors of entire HDAC classes such as I and IIa [20,21,36], and IIb [50], have all been demonstrated recently to be neuroprotective in models of PD. Notably, inhibition of the class III HDAC, Silent Information Regulator 2 (SIRT 2), has become increasingly implicated as a novel target for mediation of neuroprotection in PD [8,12,14,16,17,32,34,52]. For example, Outeiro et al. [34] have previously demonstrated that AGK2, a potent inhibitor of SIRT 2 dose dependently protects dopaminergic neurons from death in a transgenic αSyn overexpressing drosophila models of PD.

Although the neuroprotective phenotype of HDAC selective, such as SIRT 2, inhibitors have been demonstrated *in vivo* in animal models of PD, thus far pathogenic histone hypoacetylation and transcriptional dysfunction in the nigrostriatal of PD is yet to be confirmed. The acetylation level of histones within degenerating regions of the Parkinsonian brain must therefore be quantified and compared with age matched control subjects to confirm this hypothesis in the human disease and rationalise the use of HDACIs for the treatment of PD. Additionally, although it is thought that pathogenic histone hypoacetylation is in part due to the ‘masking’ effects of αSyn aggregates toward histone proteins, it remains unanswered whether the expression levels of HDACs in the brain are affected in PD. This is crucial as without confirming the maintenance of HDAC expression levels in the Parkinsonian brain, the use of HDACIs for therapeutic use in PD cannot be rationalised. Therefore here, for the first time, we quantify histone acetylation levels in the SNpc, the area known to predominantly degenerate with PD development, in brain tissue from both early (Braak stage 3/4 [3]) and late (Braak stage 6 [3]) stage PD cases, as well as age matched controls, to determine if histone acetylation is indeed a function of PD development. Additionally, given the implication of the use of HDAC (such as SIRT 2) inhibitors for neuroprotection in PD, we quantify HDAC expression in the SNpc of these same cases, to determine if the expression level of these enzymes changes with PD development. Furthermore, we seek to validate the neuroprotective and anti-inflammatory potential of SIRT 2 inhibition with the use of cell culture models of dopaminergic neurodegeneration and microglial activation, respectively, highlighting this HDAC as a potential therapeutic target for the treatment of PD.

## 2 Materials and methods

### 2.1 Human brain tissue

Human brain tissue samples (PD and aged-matched controls) were obtained from the Parkinson’s UK Tissue Bank at Imperial College London, and all experiments using the tissue samples were previously approved by the PUKTB’s Ethical Review Panel. Tissue from 10 control cases (4♂: 6♀, 82.1 ± 1.9 years), 8 early PD cases (Braak stage 3.5 ± 0.2, 4♂: 4♀, 79.3 ± 1.8 years), and 12 late PD cases (Braak stage 6 ± 0, 4♂: 4♀, 79.3 ± 1.8 years) were included for study, and data presented represents all cases studied. Cases were selected based upon the Tissue Bank’s availability of snap frozen tissue from the central region of the SNpc, in Braak stage 3-4, and Braak stage 6 PD cases, and healthy age matched controls. The suitability of tissue and sampling of the central SNpc was determined and performed by an experienced Tissue Bank technician at time of sample retrieval. Supplementary Table 1 summarises the cases used, including case by case age at death, cause of death, post-mortem delay, and for PD cases, age at disease onset, disease duration, Braak stage, and any PD medication taken in life.

### 2.2 Cell cultures

The rat mesencephalic dopaminergic 1RB3A_N27_ (N27) cell line possesses both biochemical and physiological properties of dopaminergic neurons [1], making it an ideal candidate cell line for the modelling of the Parkinsonian dopaminergic neuronal cell death *in vitro*. Prior to experimentation, N27 expression of NeuN and TH were confirmed by western blot analysis (see below for methods), confirming the dopaminergic neuronal phenotype of this cell line (supplementary Fig. 1). N27 cells (Millipore, UK) (up to passage number 45) were maintained in RPMI 1640 medium (Sigma, UK) supplemented with 10% foetal calf serum, 2 mM L-glutamine, 50U/ml Penicillin and 50 μg/ml Streptomycin (all Gibco, UK) (complete N27 medium), in a humidified incubator temperature controlled at 37 °C and with 5% CO_2_ ventilation. For experimentation, neurons were seeded into 96 well plates (Corning, UK) at a density of 10 × 10^3 cells/well, and left for 24 h to allow neurons to readopt their natural morphology. On the day of experiments cell medium was removed and replaced with fresh complete N27 medium. For induction of neurodegeneration, N27 cells were treated with lactacystin (Enzo Life Sciences, UK) (0.75 μM in distilled phosphate buffered saline (DPBS) (Sigma, UK)) for 24 h as this was shown to produce suitable robust sub-maximal levels of cytotoxicity (data not shown). For SIRT 2 inhibitor treatment, N27 cells were pre-treated with AGK2 (Tocris, UK) (in DPBS) for 24 h as this timepoint was shown produce significant hyperacetylation (supplementary Fig. 2).

The mouse microglial (N9) cell line stably retains microglial phenotypic cell surface markers, and most importantly are stringently activated upon treatment with LPS [41] making them an ideal cell line for the study of microglial activation *in vitro*. Prior to experimentation, N9 expression of Iba-1 was confirmed by western blot analysis (see below for methods), confirming the microglial phenotype of this cell line (supplementary Fig. 1). N9 cells (a kind gift from Dr Deanne Taylor) (up to passage number 45) were maintained in Dulbecco's Modified Eagle's Medium (Sigma, UK) supplemented with 5% foetal calf serum, 4 mM L-glutamine, 50U/ml Penicillin and 50 μg/ml Streptomycin (complete N9 medium), in a humidified incubator temperature controlled at 37 °C and with 5% CO_2_ ventilation. For experimentation, microglia were seeded into 6 well plates (Corning, UK) at a density of 500 × 10^3 cells/well, and left for 24 h to allow microglia to readopt their natural morphology. On the day of experiments cell medium was removed and replaced with fresh complete N9 medium (2 ml per well). For induction of microglial activation, N9 cells were treated with LPS (from Escherichia coli O111:B4, Sigma, UK) (125 ng/ml in DPBS) for 24 h as this was shown to produce significant yet sub-maximal production of both NO and TNFα (data not shown). For SIRT 2 inhibitor treatment, N9 cells were pre-treated with AGK2 (in DPBS) for 48 h as this timepoint was shown produce significant hyperacetylation (supplementary Fig. 2).

### 2.3 Protein and mRNA extraction

For extraction of protein and mRNA from human brain tissue samples, 30 mg of tissue from the brain block containing the SNpc for each case was collected into ribonuclease (RNase)-free microcentrifuge tubes (Ambion, UK). Brain tissue was homogenised in QIAzol^®^ Lysis Reagent (Qiagen, UK) using a tissue homogeniser (Ultra-Turrax T18, IKA, Germany). mRNA and protein was extracted from homogenised brain tissue using the RNeasy^®^ Plus Universal Mini Kit (Qiagen, UK) as per the manufacturer’s instructions. Isolated RNA was quantified spectrophotometrically using a NanoDrop ND-1000 spectrophotometer (Thermo Fischer Scientific, USA) and RNA purity verified by an average A_260/280_ ratio of 1.99 (range 1.97-2.01). The quantity of isolated protein was determined using the 96-well variant of the Bradford Assay (Sigma, UK): colour change determined using a 96-well plate reader (VersaMax Microplate Reader, Molecular Devices, UK) at 595 nm (A595). mRNA and protein were stored at −80 °C and −20 °C respectively until further analysis.

For extraction of protein from cell cultures, Radio-Immunoprecipitation Assay (RIPA) buffer (Sigma, UK) was used as per the manufacturer’s instructions. Briefly, after incubation of cells with the test compound(s) for the appropriate time period in a 6 well plate, the growth medium was removed and cells were washed twice with ice cold DPBS to remove any residual medium. After this cells were incubated with RIPA buffer with 1% protease inhibitor cocktail (Sigma, UK) (200 μl/well) on ice for 5mins. Cells were then scraped manually from the surface using a sterile scraper (VWR, UK) to removed and lyse residual cells. Cell lysate was collected and clarified to remove denatured nucleic acid by centrifugation and 8000 g for 10mins at 4 °C. The protein containing supernatant was then transferred to new tubes and quantified using the Bradford assay (Bradford Reagent, Sigma, UK). Proteins were stored at −20 °C until further Western blot analysis.

### 2.4 Western blot analyses

Laemmli sample buffer (Sigma, UK) was added to 10 μg extracted protein sample from either human tissue or cell cultures, and denatured by incubating at 95 °C for 15mins. Samples were loaded onto a 1 mm thick hand-cast 15% Tris-Glycine gel and proteins were separated by electrophoresis (65 mA for 40mins). Proteins were transferred onto methanol soaked PVDF membrane (Millipore, UK) with a pore size of 0.45 μm using semi-dry transfer (20 V for 45mins). Membranes were then equilibrated in TBS containing 0.2% Tween-20 (TBS-T) (Sigma, UK), before being blocked in 5% non-fat milk (Sigma, UK) in TBS-T for 1hr at room temperature. Membrane was washed in TBS-T again before being incubated in primary antibodies against either histone protein H3 acetylated on lysine 9 (rabbit anti-AcH3-Lys9, Sigma, UK, 1:10,000) and mouse anti-β-actin antibody (1:20,000, Abcam, UK) for 1hr at RT, or tyrosine hydroxylase (rabbit anti-TH, Millipore, UK, 1:1000) and NeuN (mouse anti-NeuN, Millipore, UK, 1:1000) for 20 h at RT, or Iba-1 (rabbit anti-Iba-1, Wako, Japan, 1.67 μg/ml) for 20hr at 4 °C. Membranes were then washed again and incubated in horseradish peroxidase (HRP)-conjugated secondary antibodies (either Goat anti-Rabbit [1:10,000] (for detection of rabbit anti- AcH3-Lys9, TH, and Iba-1) and Horse anti-Mouse [1:10,000] (for detection of mouse anti-β-actin, and NeuN), both Vector Laboratories, UK) for 1hr at RT. Membranes were washed again in TBS-T and developed using chemiluminescence (Clarity Western ECL Substrate, Bio-Rad, UK). Bands were quantified using densitometry analysis software (ImageJ, v1.4). For Western blot analysis of human brain derived proteins, the 30 samples were run on 3 separate gels, each gel containing samples from all three disease groups. Additionally, β-actin was used as a loading control for all samples, and AcH3-Lys9 expression shown relative to β-actin content, and as a percentage of the control subject group.

### 2.5 Quantitative real time polymerase chain reactions

For cDNA synthesis, 500 ng of total RNA from each sample was reverse transcribed according to the manufacturer's instructions using the QuantiTect^®^ reverse transcription kit (Qiagen, UK) with integrated removal of genomic DNA contamination. The reactions were stored at −20 °C until further use. Real-time reverse transcriptase quantitative polymerase chain reaction (RT-qPCR) experiments were performed using a Mx3000P™ real-time PCR system with MxPro software (v4.10, Stratagene, USA) and the Brilliant^®^ II QPCR master mix with low ROX (Agilent technologies UK Ltd, UK). For each gene of interest in each sample, 20 μl reactions were set up in triplicate, and run in duplex with a novel reference gene (XPNPEP1 [X-prolyl aminopeptidase (aminopeptidase P) 1] [15]), with each reaction containing 10 μl 2 × Brilliant^®^ II QPCR master mix, 7 μl RNase-free water, 1 μl template cDNA, 2 μl (1 μl gene of interest + 1 μl reference gene) 10 × PrimeTime™ qPCR assays (Integrated DNA technology, USA). See supplementary Table 2 for probe and primer sequences. Reactions were carried out with the following cycling protocol: 95 °C for 10 min, then 60 cycles with a 3-step program (95 °C for 30s, 55 °C for 30 s and 72 °C for 30s). Fluorescence data collection was performed during the annealing step. A negative control containing no cDNA template was also run in each plate. Similarly, an inter-plate calibrator, created by pooling control cDNA samples, was also run in each plate. Relative gene expression was determined using the 2^−DΔCT^ method normalising to the expression of the novel reference gene [15] and the appropriate control group.

### 2.6 Cell viability assays

#### 2.6.1 MTS assay

For performing the MTS assay the CellTiter 96^®^ AQueous One Solution Cell Proliferation Assay Kit (Promega, USA) was used as per the manufacturer’s instructions. Briefly, after incubation of cells with the test compound(s) for the appropriate time period in a 96 well plate in triplicate, 20 μl CellTiter 96^®^ AQueous One Solution reagent was added directly to each well containing 100 μl cell culture medium. Plates were then incubated at 37 °C in a humidified incubator temperature with 5% CO_2_ ventilation for 3 h. Absorbance was read using a 96-well plate reader at 490 nm (A490). The absorbance at 490 nm (A490) was then converted to% of control group and data expressed as mean ± standard error or mean (SEM).

#### 2.6.2 Neutral red assay

For performing the NR assay a previously published protocol was followed [40]. Briefly, NR stock solution (4 mg/ml NR dye (Sigma, UK) in DPBS) was dissolved into complete cell culture medium (either N9 or N27 depending on the cell line tested) to give a final concentration of 40 μg/ml NR (henceforth referred to as NR medium) and incubated at 37 °C for 24 h. To perform the assay, after incubation of cells with the test compound(s) for the appropriate time period in a 96 well plate in triplicate, the test medium was removed by aspiration and replaced with 100 μl neutral red medium. Plates were incubated at 37 °C in a humidified incubator temperature controlled at 37 °C and with 5% CO_2_ ventilation for 3 h. After this time cells were inspected using an inverted microscope to confirm intracellular precipitation of NR. The NR medium was removed by aspiration from wells and cells washed by adding 150 μl DPBS and removing by aspiration. 150 μl NR destain solution (50% ethanol, 49% water, 1% glacial acetic acid (all Sigma, UK)) was added to wells before plates were shaken rapidly on a microtitre plate shaker for 10mins to solubilise the dye. Absorbance was read using a 96-well plate reader at 540 nm (A540). The absorbance at 540 nm (A_540_) was converted to% of control group and data expressed as mean ± SEM.

#### 2.6.3 Bradford assay

Briefly, after spectrophotometric reading of the wells for the NR assay, the neutral red destain solution was removed from wells by aspiration before cells were washed three times with 150 μl DPBS. Cells were then lysed and proteins solubilised by addition of 50 μl sodium hydroxide (0.1 M) (Sigma, UK) solution. Protein standards were run in parallel with each plate by addition of 50 μl sodium hydroxide (0.1 M) solution to 5 μl of a known concentration of protein (0 − 1.4 mg/ml BSA (Sigma, UK) in dH_2_0) run in triplicate. 200 μl Bradford Reagent was added to each well and plates were shaken rapidly on a microtitre plate shaker for 20mins. Absorbance was read using a 96-well plate reader at 595 nm (A595). A standard curve of the spectrophotometric reading at A_595_ for each standard, minus the reading for the standard void of protein was plotted against its protein concentration. The slope of the line of best fit of this data set was used for calculation of the protein concentration for cell lysates. Data was converted to% of control group and data expressed as mean ± SEM.

#### 2.6.4 Microglial activation assays

##### 2.6.4.1 Griess assay

In biological systems, NO is auto-oxidised into two stable metabolites: nitrite (NO_2_^−^) and nitrate (NO_3_^−^). The Griess Assay was therefore used for determination of one of these metabolites, nitrite, as an indicator of NO production. For performing the Griess assay, after incubation of microglial cells with the test compound(s) for the appropriate time period in a 6 well plate, the medium was removed and centrifuged at 1200*g* for 5mins to removed cell debris. 100 μl of medium samples were pipetted into a 96 well plate in triplicate. In parallel, standards of known nitrite concentration (0 − 50 μM sodium nitrite (Sigma, UK) in complete N9 medium) were run in triplicate in order to translate the spectrophotometric reading to nitrite concentration. 100 μl Griess Reagent (Sigma, UK) was added directly to each well and plates were shaken rapidly on a microtitre plate shaker for 10mins. Absorbance was read using a 96-well plate reader at 540 nm (A540). A standard curve of the spectrophotometric reading at A_540_ for each standard, minus the reading for the standard void of nitrite was plotted against its nitrite concentration. The slope of the line of best fit of this data set was used for calculation of the nitrite concentration for medium samples. Data was converted to% of control group and data expressed as mean ± SEM.

##### 2.6.4.2 TNFα enzyme-linked immunosorbant assays

For performing TNFα ELISAs, murine TNFα ELISA development kits (Peprotech Ltd., UK) were used as per the manufacturer’s instructions. Briefly, the surface of a high binding EIR/RIA 96 well plate (Corning, UK) was coated with the anti-TNFα capture antibody (1 μg/ml in PBS) overnight at RT. After this time, wells were washed four times in PBST (PBS with 0.05% Tween-20). Non-specific binding was then blocked by incubating wells with PBS with 1% BSA for 1hr at RT. Wells were then washed again four times with PBST and incubated with either sample or standard (0–2 ng/ml TNFα in PBS with 0.05% Tween-20 and 0.1% BSA) run in triplicate and incubated for 2 h at RT. Wells were then washed again four times in PBST and incubated with anti-TNFα detection antibody (0.25 μg/ml) for 2 h at RT. Wells were washed again four times in PBST and incubated in avidin complex (1:2000 TNFα in PBS with 0.05% Tween-20 and 0.1% BSA) for 30mins at RT. Wells were washed a final four times in PBST and 100 μl of ABTS added to each well. Absorbance was read using a 96-well plate reader at 405 nm and 650 nm (A405 and A650, respectively). Colour development was monitored for ∼45mins and the reading in which the A_405nm_ was ≤0.2 for the 0 ng/ml TNFα standard and ≤1.4 for the 2 ng/ml TNFα as per the manufacturer’s instruction. A standard curve of the spectrophotometric reading at A405 minus the reading at A650 was plotted against TNFα. The equation of this line of best fit was then used for calculation of the TNFα concentration for medium samples. Data was then converted to% of control group and data expressed as mean ± SEM.

### 2.7 Statistics

A one way ANOVA with Bonferroni post-test was used to compare AcH3-Lys9 expression between human control and PD cases. Linear regression analyses were used to test correlations between data. A two-way ANOVA with Bonferroni post-test was used to compare TH and HLA-DPα1 expression, and HDAC expressions between human control and PD cases. Unpaired *t*-tests were used for comparison of treated cell lines to vehicle treated lines. Lastly, a one-way ANOVA with Dunnett post-test was used to compare AGK2-treated cell lines to vehicle treated lines. All data, unless otherwise stated, is presented as mean ± standard error of mean, from all human cases described (supplementary Table 1) or n = 3 independent repeats for cell culture experiments. All statistical tests were performed using GraphPad Prism (v5.0 for Windows, GraphPad Software, San Diego, CA, USA).

## 3 Results

### 3.1 Histone acetylation in the SNpc of the human brain during PD development

**Fig. 1:**
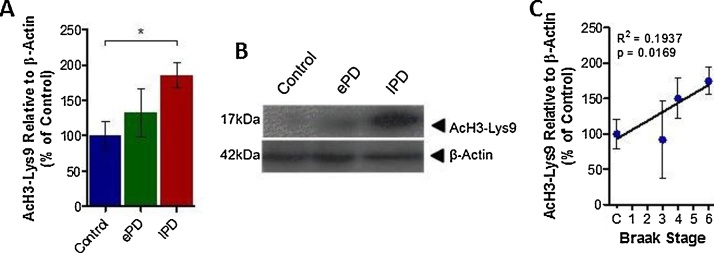
Histone Acetylation and Parkinson’s Disease Progression. Histone acetylation (AcH3-Lys9) was quantified in the Substantia Nigra pars compacta (SNpc) of each control, each early, and each late PD case relative to β-Actin using Western blot analysis. (A) Bar graph illustrating level of AcH3-Lys9 observed in each group of cases. (B) Representative blot of data presented in (A). (C) Graphical representation of correlation between Braak Stage of human cases and AcH3-Lys9 observed in the SNpc of that case. N = 8-12 per group (see supplementary Table 1). Statistical significance indicated with asterisks: *p < .05. Abbreviations: ePD, early Parkinson’s disease; lPD, late Parkinson’s disease.

Quantification of the commonly acetylated histone residue, AcH3-Lys9, in protein extracts from the SNpc of control, early, and late PD cases (see supplementary Table 1) revealed a disease-dependent increase in acetylation of this residue on histone 3 in this brain region. There was a subtle increase in AcH3-Lys9 observed from control in early PD cases and significantly more AcH3-Lys9 from control in late stage PD cases (Fig. 1A and B, early and late PD cases, 132.70 ± 34.16% and 185.11 ± 17.61% of control respectively, p > .05 and p < .05). Correlation analysis between the level of histone acetylation and the Braak stage of each case revealed a significant correlation between these two measures, indicative of histone acetylation in the SNpc with PD development (Fig. 1C, R^2^ = 0.1937, p < .05).

### 3.2 Changes in expression of TH, HLA-DPα1 and SIRT 2 in the SNpc with PD development

**Fig. 2:**
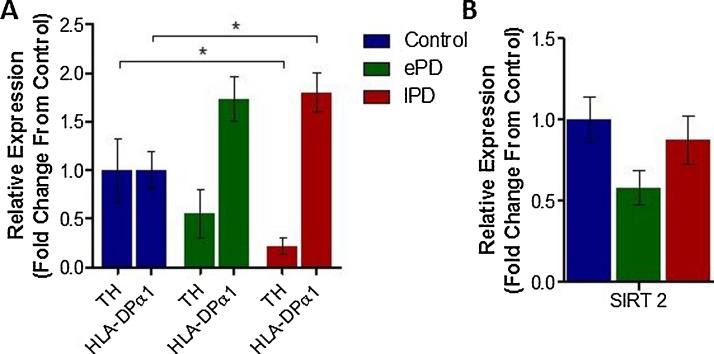
Relative mRNA Expression of TH, HLA-DPα1 and SIRT 2 in Control and PD SNpc Tissue Samples. (A) mRNA expression levels of cellular markers TH and HLA-DPα1 in the SNpc of control and PD cases, relative to a housekeeping gene expression, quantified using qRT-PCR. (B) mRNA expression levels of the HDAC SIRT 2 in the same cases. N = 8-12 per group (see supplementary Table 1). Statistical significance indicated with asterisks: *p < .05. Abbreviations: TH, tyrosine hydroxylase; HLA-PDα, Human Leukocyte Antigen DPα1, SIRT 2, Silent Information Regulator 2; ePD, early Parkinson’s disease; lPD, late Parkinson’s disease.

As would be expected, the mRNA expression level of tyrosine hydroxylase (TH), the rate limiting enzyme in monoamine synthesis, was disease-dependently reduced, in line with degeneration of dopaminergic neurons in the SNpc in PD (Fig. 2, early and late PD cases, 0.55 ± 0.25 and 0.22 ± 0.09 fold change from control respectively, p > .05 and p < .05). Correlation analysis between the level of SNpc mRNA TH expression and the Braak stage of each case revealed a significant negative correlation between these two measures (Table 1, R^2^ = 0.2216, p < .01). The opposite was true for expression of Human Leukocyte Antigen DPα1 (HLA-DPα1), a MHC protein known to be present on activated infiltrating microglia: a disease-dependent increase in HLA-DPα1 mRNA expression being observed with PD development (Fig. 2, early and late PD cases, 1.73 ± 0.23 and 1.80 ± 0.20 fold change from control respectively, p > .05 and p < .05). Correlation analysis between the level of SNpc HLA-DPα1 mRNA expression and the Braak stage of each case revealed a significant positive correlation between these two measures (Table 1, R^2^ = 0.1585, p < .05). Interestingly however, probably due to the large amount of TH positive cell death with disease development, histone acetylation (AcH3-Lys9) correlated only with HLA-DPα1 mRNA expression in the SNpc of diseased brain tissue (Table 1, R^2^ = 0.2951, p < .01), not TH mRNA expression.

**Table 1:** Results of Regression Analyses Performed Between TH, HLA-DPα, SIRT2 Expression and Braak Stage and AcH3-Lys9 Expression.

Correlation		Slope	R^2^	P Value	
Braak Stage vs.	TH	−0.1311 ± 0.04641	0.2216	0.0086	**
	HLA-DPα	0.1384 ± 0.06649	0.1585	0.0487	*
	SIRT 2	−0.02307 ± 0.03272	0.01744	0.4866	
AcH3-Lys9 vs.	TH	−0.001152 ± 0.001835	0.01438	0.5355	
	HLA-DPα	0.006618 ± 0.002181	0.2951	0.0061	**
	SIRT 2	2.038e-005 ± 0.001156	0.00001151	0.9861	

Abbreviations: TH, tyrosine hydroxylase; HLA-PDα, Human Leukocyte Antigen DPα1, SIRT 2, Silent Information Regulator 2. N = 8-12 per group (see supplementary Table 1).

The expression levels of HDACs 1-10, and SIRTs 1 and 2, did not follow such a disease-dependent pattern: revealing no statistically significant changes in expression between either early or late PD cases compared from controls (Table 2). Of note, SIRT 2 expression in the early PD cases was 41.80 ± 10.67% reduced compared to control cases, however in late stage PD cases SIRT 2 expression was reduced by only 12.53% ± 14.74% (Fig. 2B). Correspondingly, SIRT 2 expression did not correlate with either Braak staging of cases or the level of histone acetylation, indicative of a more complex mechanism of disease-dependent change (Table 1). Comparable levels of infiltrating microglia (HLA-DPα1 expression) between early and late stage cases, combined with exacerbated levels of dopaminergic neurodegeneration (TH expression) in late stage cases compared to early however could explain this pattern of change.

**Table 2:** Results of Changes in Expression of HDACs in the SNpc of Control and PD cases.

Class	Isoform	Relative Expression (Fold Change From Control)			
		Early PD		Late PD	
		Mean ± SEM	P Value	Mean ± SEM	P Value
I	HDAC1	1.2357 ± 0.1152	>0.05	1.0550 ± 0.1852	>0.05
	HDAC2	1.2852 ± 0.2858	>0.05	1.1276 ± 0.1603	>0.05
	HDAC3	0.4194 ± 0.0387	>0.05	0.5014 ± 0.0673	>0.05
	HDAC8	0.6861 ± 0.1515	>0.05	1.2577 ± 0.2154	>0.05
IIa	HDAC4	1.0481 ± 0.2123	>0.05	0.8516 ± 0.2084	>0.05
	HDAC5	0.3724 ± 0.2360	>0.05	0.7682 ± 0.1731	>0.05
	HDAC7	0.7703 ± 0.1198	>0.05	0.4296 ± 0.0700	>0.05
	HDAC9	1.0882 ± 0.1929	>0.05	0.8917 ± 0.1041	>0.05
IIb	HDAC6	1.3227 ± 0.2378	>0.05	0.7030 ± 0.1087	>0.05
	HDAC10	1.0997 ± 0.2442	>0.05	0.7087 ± 0.1079	>0.05
III	SIRT1	1.0097 ± 0.1486	>0.05	0.8456 ± 0.0556	>0.05
	SIRT2	0.5819 ± 0.1067	>0.05	0.8747 ± 0.1473	>0.05

Abbreviations: HDAChistone deacetylase; PDParkinson’s disease; SEMstandard error of mean. N = 8–12 per group (see supplementary Table 1).

### 3.3 Histone acetylation levels in degenerating dopaminergic neurons and activated microglia in culture reveal opposing effects of pathology

**Fig. 3:**
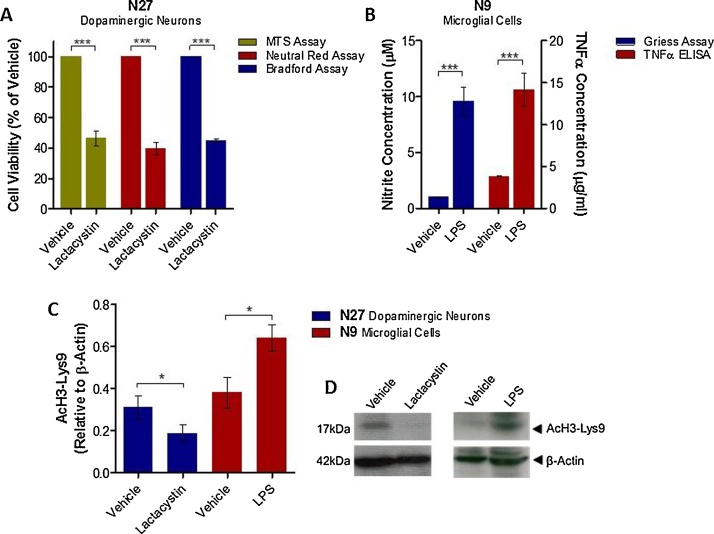
Histone Acetylation Levels in Degenerating Dopaminergic Neuron and Microglial Cell Cultures. (A) Degeneration of dopaminergic neuronal cultures induced by lactacystin was confirmed using cell viability assays (MTS, neutral red, and Bradford assays). (B) Activation of microglial cultures induced by LPS was confirmed using assays for TNFα and NO. Histone acetylation (AcH3-Lys) was quantified in degenerating N27 dopaminergic neuronal cultures and N9 microglial cultures relative to β-Actin using Western blot analysis. (C) Level of AcH3-Lys9 observed with each treatment. (D) Representative blot of data presented in (C). N = 3 independent replicates. Statistical significance indicated with asterisks: *p < .05, ***p < .001.

To isolate the effects of neurodegeneration through altered protein accumulation on histone acetylation in dopaminergic neurons, and the effects of microglial activation on histone acetylation, cell culture systems were utilised. Incubation of N27 mesencephalic dopaminergic neurons with the irreversible ubiquitin proteasome inhibitor, lactacystin, for 24 h induced a significant degree of neurodegeneration, cell viability quantified through the MTS, neutral red, and Bradford assays (Fig. 3A, lactacystin vs. vehicle treated neurons, p < .001 in all three assays). Accompanying this, degenerating dopaminergic N27 neurons were observed to have a significantly decreased level of histone acetylation compared to vehicle treated neurons (Fig. 3C and D, AcH3-Lys9 relative to β-Actin, 0.31 ± 0.05 in vehicle treated vs. 0.19 ± 0.04 in lactacystin treated neurons, p < .05). Incubation of N9 microglial cells with lipopolysaccharide (LPS) for 24 h induced a significant degree of microglial activation, indirectly measured through quantification of NO and TNFα secreted into the cell culture medium (Fig. 3B, LPS vs. vehicle treated microglia, p < .001 in both assays). Accompanying this, activated N9 microglia were observed to have a significant increase in histone acetylation compared to vehicle treated microglia (Fig. 3, AcH3-Lys9 relative to β-Actin, 0.38 ± 0.07 in vehicle treated vs. 0.64 ± 0.06 in lactacystin treated neurons, p < .05). These findings are therefore in agreement with those above, in which a strong correlation between histone acetylation and PD development was observed, in line with both a disease-dependent reduction in dopaminergic neuron marker and increase in activated microglial marker.

### 3.4 SIRT 2 inhibition translates to neuroprotection in neuronal cell cultures

**Fig. 4:**
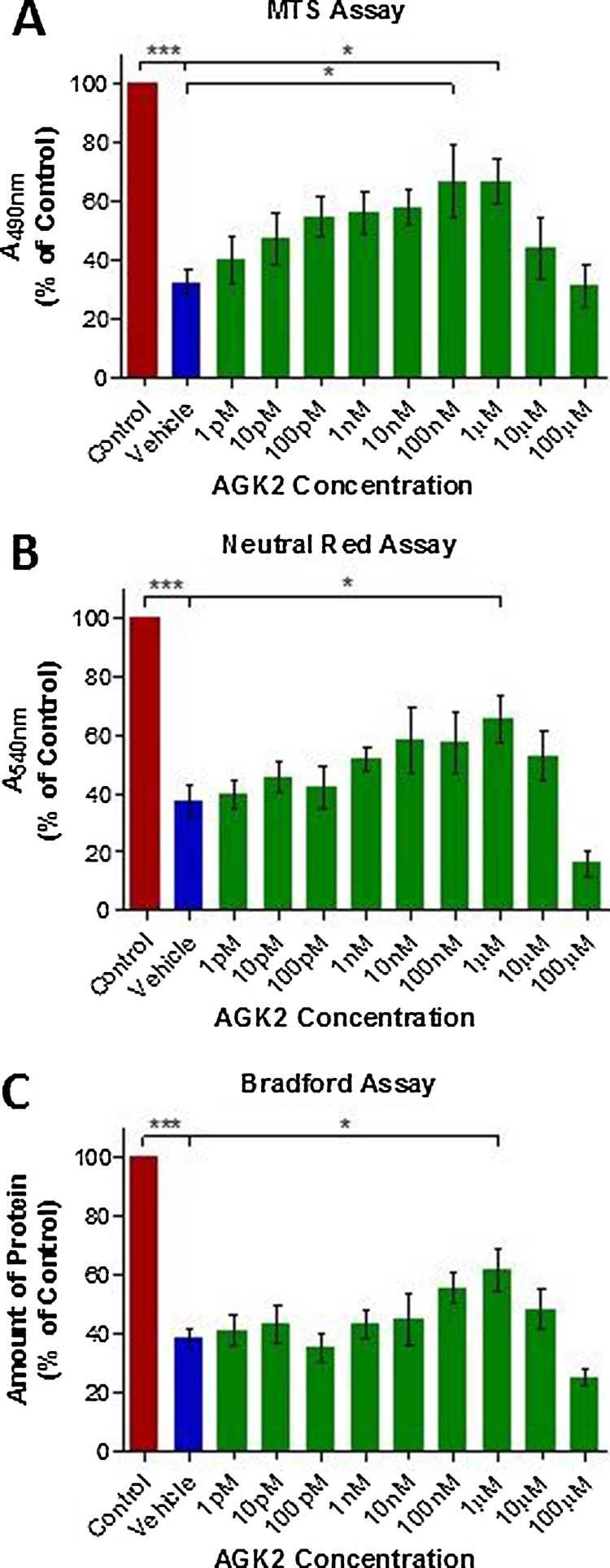
Neuroprotective Effects of SIRT 2 Inhibition via AGK2 Treatment of N27Cells. Cells were pre-treated with AGK2 (or vehicle) for 48 h prior to addition of lactacystin (0.75 μM or vehicle) for a further 24 h. (A) MTS, (B) neutral red, and (C) Bradford assays were then performed for quantification of AGK2 induced neuroprotection against lactacystin toxicity. Red bars indicate cells which received vehicle in place of both AGK2 and lactacystin. Blue bars indicate cells which received vehicle in place of AGK2 treatment but subsequently received lactacystin treatment. Green bars indicate cells treated with AGK2 and which subsequently received lactacystin treatment. N = 3 independent replicates. Statistical significance indicated with asterisks: *p < .05, ***p < .001.

Neurodegeneration of dopaminergic N27 neurons exerted by lactacystin (0.75 μM), has been shown above to produce significant histone hypoacetylation. Therefore to determine whether or not histone acetylation through SIRT 2 inhibition would translate to neuroprotection *in vitro*, dopaminergic N27 cells were treated with a range of concentrations (1pM to 100 μM) of AGK2, a potent SIRT 2 inhibitor, prior to treatment with lactacystin (0.75 μM) to cause degeneration. Cell viability was then quantified using MTS, Neutral Red, and Bradford assays. In all three viability assays neurotoxicity was observed at AGK2 concentrations ≥10 μM (Fig. 4A, B and C). However, treatment of N27 neurons with 1 μM AGK2 prior to treatment with lactacystin resulted in a significant increase in cell viability compared with vehicle treated cells, in all three assays (Fig. 4A, B and C, 1 μM AGK2, p < .05 for each assay).

### 3.5 SIRT 2 inhibition translates to anti-inflammation in microglial cell cultures

**Fig. 5:**
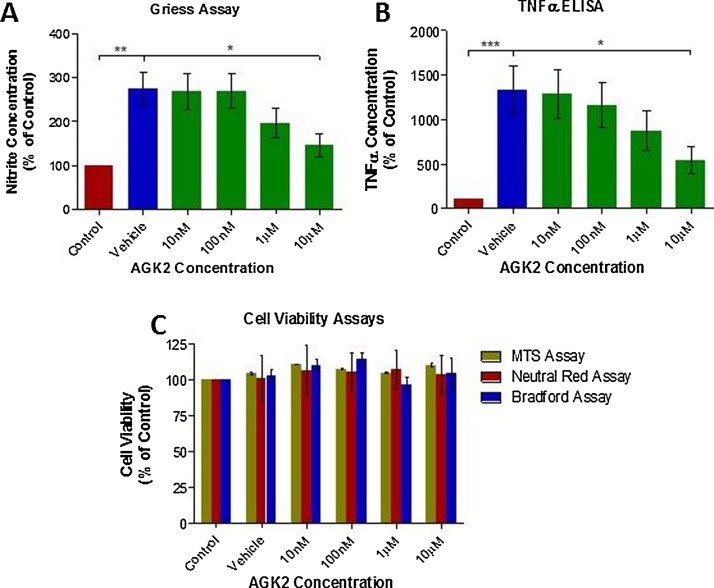
Anti-Inflammatory Effects of SIRT 2 Inhibition via AGK2 Treatment of N9Cells. Cells were pre-treated with AGK2 (or vehicle) for 24 h prior to addition of LPS (125 ng/ml or vehicle) for a further 24 h. (A) Griess Assay and (B) TNFα ELISAs were then performed on the medium for quantification of AGK2 induced reduction of LPS induced microglial activation. Red bars indicate cells which received vehicle in place of both AGK2 and LPS. Blue bars indicate cells which received vehicle in place of AGK2 treatment but subsequently received LPS treatment. Green bars indicate cells treated with AGK2 and which received subsequently received LPS treatment. (C) Cell viability assays of each treatment of N9 cells. N = 3 independent replicates. Statistical significance indicated with asterisks: *p < .05, **p < .01, ***p < .001.

Activation of N9 microglial cells through treatment with LPS (125 ng/ml), has been shown above to produce significant histone hyperacetylation. To determine the effects of SIRT 2 inhibition on microglial activation, N9 microglia were treated with a range of concentrations (10 nM to 10 μM) of AGK2, prior to treatment with LPS (125 ng/ml) to trigger activation. Quantification of secreted NO and TNFα from N9 microglia were used as surrogate markers of activation as a result of LPS treatment. Cell culture medium concentrations of both NO and TNFα were observed to be significantly reduced in N9 microglia treated with 10 μM AGK2 compared with vehicle treatment (Fig. 5A and B, p < .05 in both assays), indicative of reduced microglial activation at this concentration. To confirm that the observed reductions in NO and TNFα were indeed a result of reduced microglial activation and not cytotoxicity, cell viability after AGK2 and LPS treatment was quantified using MTS, Neutral Red, and Bradford assays. No cytotoxicity was observed at any concentration of AGK2 tested (Fig. 4C).

## 4 Discussion

Here we have demonstrated for the first time that histone acetylation within the SNpc positively correlates with PD pathological development in the human brain, which is associated with neurodegeneration of dopaminergic neurons. This is in contrast to our *in vitro* findings, in which histone hypoacetylation was observed in degenerating dopaminergic neurons following treatment with lactacystin. This dichotomy could potentially be explained by the infiltration of activated microglia into the degenerating SNpc in PD. Indeed we have subsequently demonstrated that microglial activation is associated with a marked increase in histone acetylation, *in vitro*, and the extent of histone acetylation observed in the human brain correlates closely with the degree of the microglial marker, HLA-DPα1, and the Braak stage of the disease. Furthermore, the neuroprotective and anti-inflammatory effects of SIRT 2 inhibition *in vitro*, and the maintained expression level of this HDAC in the degenerating SNpc in the Parkinsonian brain, highlight the therapeutic potential of targeting this HDAC for disease modification in PD.

From previous studies in cell culture systems and animal models of neurodegenerative disease, it has been described that there is an imbalance between the activities of HATs and HDACs in neurodegeneration, in favour of histone deacetylation, thought to play a role in pathogenesis and disease progression [13,43,45]. Furthermore, it has described that αSyn accumulation itself promotes histone hypoacetylation, thought to be achieved through αSyn ‘masking’ acetylation sites on histone proteins [31]. Here, in a degenerating region of the human brain, we report the opposite: that PD development is associated with histone hyperacetylation rather than hypoacetylation in the SNpc. It is important to note that in previous studies, these observations were made in homogenous populations of brain cells, for example, dopaminergic SH-Sy5Y cells alone in culture overexpressing αSyn in the study by Kontopoulos et al. [31], rather than in brain tissue containing not only dopaminergic neurons but non-neuronal brain cells too. Likewise, our *in vitro* findings of dopaminergic N27 neurons alone in culture, treated with lactacystin, are in agreement with those published previously [31], corroborating that neurodegeneration likely induces histone hypoacetylation in dopaminergic neurons. In the dopaminergic SNpc in humans however, here we observe a positive correlation of histone acetylation with Braak staging of disease rather than a negative one. Combined with this however we also observe a disease-dependent increase in marker expression levels of activated microglia. In culture, when activated, microglia displayed histone hyperacetylation. Therefore, it is likely that in the Parkinsonian brain as the disease develops, the reduction of dopaminergic neurons expressing histone hypoacetylation, combined with the infiltration of activated microglia expressing histone hyperacetylation, results in a net disease-dependent increase in the level of histone acetylation. Our correlation analyses add weight to this proposed mechanism: histone acetylation correlating with microglial marker expression directly, yet not with dopaminergic neuronal marker expression, likely due to the extent of dopaminergic neurodegeneration and microglial infiltration with the development of PD.

In the current study, mRNA and protein were extracted from whole brain tissue of the SNpc. And as has been described above, due to the differences in cell composition (ratio of dopaminergic neurons to microglia) between control, early and late PD cases, it makes interpretation of data from such a disease-dependently varied region difficult. Cell culture studies of individual neuronal and microglial populations were utilised in order to aid interpretation, but a number of caveats come with the use of immortalised animal cell lines, when comparing to data from the human brain. In contrast, isolation of select cell populations from the human SNpc, with the use of techniques such as laser capture microdissection (LCM) would eliminate such issues, and as such could provide direct quantification of measures such as histone acetylation and SIRT 2 expression, in degenerating neurons or activated microglia in the SNpc. Further work should therefore seek to confirm the findings from degenerating dopaminergic neurons and activated microglia *in vitro*, in select cell populations in the human SNpc using techniques such as LCM.

In dopaminergic neurons in PD, a misbalance between the activities of HATs/HDACs in favour of histone deacetylation is thought to result in neurodegeneration due to dysregulation of appropriate gene expression and subsequent failure of neuronal homeostasis [13,43,45]. Consistently, here we have demonstrated that neurodegeneration of dopaminergic neurons *in vitro*, is indeed accompanied by histone hypoacetylation. Furthermore, previous studies suggest that the observed pathogenic histone hypoacetylation in degenerating dopaminergic neurons can be rectified by specifically inhibiting the SIRT 2 HDAC. Reliably, pre-treatment of N27 cells in the current study with a potent SIRT 2 inhibitor, AGK2, resulted in a significant degree of neuroprotection at appropriate concentrations. Cytotoxicity however was observed at higher concentrations of AGK2, perhaps due to excessive histone hyperacetylation as a result of AGK2 treatment, however this has not been directly quantified in the current study. Likewise, it has not been ascertained in the current study as to what degree the histone deacetylation in degenerating neurons was rectified upon treatment with AGK2, that would result in neuroprotection in appropriate concentrations of AGK2. Similarly, HDACIs are thought to act neuroprotectively via a number of different mechanisms, the most well understood is their rectification of neuronal homeostasis via upregulation of neurotrophic and anti-apoptotic factors [22,47]. For example, we showed recently that HDACI treatment in an animal model of PD results in neuroprotection, associated with upregulation of brain derived neurotrophic factor (BDNF), glial derived neurotrophic factor (GDNF), and anti-apoptotic factor B-Cell Lymphoma 2 (BCL-2) [21]. The mechanisms mediating the neuroprotective effects observed in dopaminergic neurons in the current study have not been investigated here, therefore further work should aim to understand the specific neuroprotective mechanism of SIRT 2 inhibition in this cell type, through quantification of such factors along with quantification of histone acetylation, in treated degenerating neurons. In addition to further investigation of the neuroprotective mechanism of SIRT 2 inhibition observed in dopaminergic neurons here, it is important to consider whether or not the effects are relevant to the specific phenotype of dopaminergic neurodegeneration observed in PD. For example, lactacystin, through its inhibition of the ubiquitin proteasome system, is known to inhibit Nuclear Factor Kappa B (NFкB) [18], the opposite of what is observed in dopaminergic neurons of PD patients [24]. Therefore to determine whether SIRT 2 inhibition is likely to have a similar neuroprotective phenotype to that observed here in lactacystin treated dopaminergic neurons, in degenerating dopaminergic neurons in PD, further work should aim to repeat the experiments in neuronal cultures, with additional neurodegenerative compounds (such as 6-hydroxydopamine (6-OHDA), or 1-methyl-4-phenylpyridinium (MPP + )) modelling additional facets of the profile of neurodegeneration displayed by dopaminergic neurons in PD.

When cultured and treated with LPS, microglia display a multi-faceted profile of activation, adopting an activated amoeboid morphology, upregulating pro-inflammatory cytokines such as TNFα, IL6 and IL1β, and activation of iNOS resulting in production of NO [33]. It is hypothesised that upregulation of these numerous factors results from transcriptional upregulation, via histone acetylation and chromatin relaxation. Correspondingly, consistent with similar observations in other microglial cell lines [39], we demonstrate here that histone acetylation is increased when N9 microglial cells are treated with LPS, along with increased secretion of TNFα and NO. It would therefore appear counterintuitive that a HDAC inhibiting agent, which increases histone acetylation and gene transcription, would result in amelioration of microglial activation. Likewise, non-selective HDACIs Trichostatin A and Suberoylanilide Hydroxamic acid have been previously shown to potentiate activation and cytokine secretion in N9 microglia [51]. In primary mouse and human microglia however, the opposite has been described, that such non-selective HDACIs attenuate microglial activation and production of pro-inflammatory cytokines and NO [28,44,49]. Neither of these latter studies however confirm microglial cell viability following LPS and HDACI treatment, and given findings from Jau-Shyong Hong’s laboratory that non-selective HDACI such as valproate attenuate LPS induced dopaminergic neurotoxicity through HDACI mediated microglial apoptosis [6,37], it may well be likely that the reduced secretion of pro-inflammatory cytokines and NO in previous studies is due to HDACI mediated apoptosis of microglial cells. In contrast however, here we describe that inhibition of a select HDAC, SIRT 2, results in attenuation of microglial activation as measured through production of TNFα and NO. Along with this, microglial apoptosis was confirmed not to be involved: cell viability of microglial cells after treatment with AGK2 and LPS shown not to be reduced. This is suggestive that SIRT 2 plays a more crucial role in activation of microglia than its effects as a HDAC enzyme. SIRT 2 has been previously shown to be the most abundant HDAC endogenously expressed by microglia, which is also stringently upregulated upon activation [28], and as such has been shown to be crucially required for LPS induced activation of microglial cells in culture [4]. Contrastingly, it was shown in 2013 that genetic deletion of SIRT 2 resulted in activation in the N9 microglial line, opposing the results shown here [35]. It may be the case that in this genetic ablation study, other HDACs are upregulated in compensation for the loss of HDAC activity: SIRT 2 being the most abundant HDAC expressed by microglia [28]. Indeed, SIRT 2 acts as a mitotic check point, which, during mitosis, is shuttled from its location in the cytoplasm to the nucleus [25]. It may therefore be that SIRT 2 inhibition reduces microglial activation by direct inhibition of replication. Additionally, when microglia are activated with toll like receptor ligands such as LPS, NFкB in the cytoplasm becomes dissociated from inhibitors such as IкB, allowing its translocation to the nucleus and facilitation of NFкB dependent transcription of inflammatory genes [35]. Further to this, SIRT 2 interacts with the p65 subunit of NFкB, deacetylating it at lysine 301 and thus enhancing its DNA binding capacity [30,42]. It is conceivable then that the reduction of microglial activation, via SIRT 2 inhibition by AGK2, may be achieved through inhibition of this mechanism rather than exacerbating the level of histone acetylation observed upon activation. That being, that SIRT 2 inhibition would indeed result in histone hyperacetylation, leading to greater access for transcription factors such as NFкB. However, the reduced DNA binding capacity of NFкB induced as a result of SIRT 2 inhibition would lead to an overall reduced level of NFкB-dependent transcription of inflammatory genes such as TNFα, which has been shown here. Indeed the difference in mechanisms for mediation of SIRT 2 inhibition in neurons and microglia may account for the disparity between efficacious doses in the two cell types, observed here *in vitro*: 1 μM in N27 dopaminergic neurons, yet 10 μM in N9 microglia. Along with the neuroprotective phenotype observed towards degenerating dopaminergic neurons, our findings on the use of a SIRT 2 inhibitor in microglia highlights the advantage of more selective HDAC inhibition, more specifically SIRT 2 inhibition, for therapeutic potential in PD.

Given the extent of histone hyperacetylation in activated microglia, and histone hypoacetylation in degenerating dopaminergic neurons, the disease-dependent change observed here with PD development can be largely accounted for. However, the contribution of astrocytes to the level of histone acetylation has yet to be considered. Astrogliosis in the SNpc in PD is a key feature of the disease [23], and as such histone hyperacetylation in such reactive glia would surely contribute to the levels of histone acetylation observed here in human SNpc tissue. Importantly however, HDACI treatment of astrocytes has been shown to increase expression and secretion levels of neurotrophic factors such as BDNF and GDNF [5,7,54], resulting in reduced neurotoxicity, achieved through increased promoter activity via promoter-associated histone acetylation [54]. Additionally, HDACI treatment of astrocytes has been noted to be associated with increased glutamate transport [53], likely to contribute to the proposed mechanism of neuroprotection of HDACIs in PD [22]. Furthermore, SIRT 2 inhibition specifically with AGK2 has been observed to reduce neuroinflammation, via downregulation of iNOS and Cyclooxygenase 2 (COX2) [46]. It is likely then that astrocytes contribute positively to the neuroprotective phenotype observed by HDACIs in animal models of PD. Likewise, the published effect of reduced inflammation in astrocytes treated with SIRT 2 inhibitors suggest that along with the effects of SIRT 2 inhibition in neurons and microglia, astrocytes would contribute to the proposed beneficial effects of SIRT 2 inhibitors for therapeutic potential in PD.

## 5 Conclusions

We have observed here using a combination of post-mortem brain tissue and cell culture systems, that along with dopaminergic neurodegeneration and microglial activation, PD development is associated with histone hyperacetylation. Further to this, we have demonstrated that SNpc expression levels of the HDAC SIRT 2 remain relatively unaltered with PD development highlighting the potential of its targeting in PD patients. Cell culture studies in which SIRT 2 was pharmacologically inhibited via treatment with AGK2, demonstrate that drug treatment results in both neuroprotection in degenerating dopaminergic neurons, and reduced activation in microglia. Taken together, our findings demonstrate that SIRT 2 inhibitors warrant further investigation as potential therapeutics for the treatment of PD. More importantly, our results highlight the need for more selective, potent and CNS penetrant SIRT 2 inhibitors to be developed, hence enabling the neuroprotective potential of this drug class to be fully assessed *in vivo* in animal models of PD, whilst avoiding any toxic effects of high drug concentrations.

## Authorship contribution statement

IFH contributed to the design of the study, performed all experiments, and wrote the manuscript. ADW performed a subset of the neuronal cell culture experiments and analysis, and contributed to the manuscript critique. DTD conceived and helped design the study, and contributed to the manuscript critique.

## Conflicting interest statement

The authors declare that they have no competing interests.

## Funding and acknowledgements

IFH was supported by a UK Medical Research Council PhD Studentship. Tissue samples and associated clinical and neuropathological data were supplied by the Parkinson's UK Tissue Bank, funded by Parkinson’s UK, a charity registered in England and Wales (258197) and in Scotland (SC037554). The authors would like to thank all the tissue donors and their families.

## References

[1] Adams F., Rosa F., Kumar S., Edwards-Prasad J., Kentroti S., Vernadakis A., Freed C., Prasad K. (1996). Characterization and transplantation of two neuronal cell lines with dopaminergic properties. Neurochem. Res..

[2] Ammal Kaidery N., Tarannum S., Thomas B. (2013). Epigenetic landscape of parkinson’s disease: emerging role in disease mechanisms and therapeutic modalities. Neurotherapeutics.

[3] Braak H., Tredici K.D., Rüb U., de Vos R.A.I., Jansen Steur E.N.H., Braak E. (2003). Staging of brain pathology related to sporadic Parkinson’s disease. Neurobiol. Aging.

[4] Chen H., Wu D., Ding X., Ying W. (2015). SIRT2 is required for lipopolysaccharide-induced activation of BV2 microglia. Neuroreport.

[5] Chen P.S., Peng G.S., Li G., Yang S., Wu X., Wang C.C., Wilson B., Lu R.B., Gean P.W., Chuang D.M., Hong J.S. (2006). Valproate protects dopaminergic neurons in midbrain neuron/glia cultures by stimulating the release of neurotrophic factors from astrocytes. Mol. Psychiatry.

[6] Chen P.S., Wang C.C., Bortner C.D., Peng G.S., Wu X., Pang H., Lu R.B., Gean P.W., Chuang D.M., Hong J.S. (2007). Valproic acid and other histone deacetylase inhibitors induce microglial apoptosis and attenuate lipopolysaccharide-induced dopaminergic neurotoxicity. Neuroscience.

[7] Chen S.H., Wu H.M., Ossola B., Schendzielorz N., Wilson B.C., Chu C.H., Chen S.L., Wang Q., Zhang D., Qian L., Li X., Hong J.S., Lu R.B. (2012). Suberoylanilide hydroxamic acid a histone deacetylase inhibitor, protects dopaminergic neurons from neurotoxin-induced damage. Br. J. Pharmacol..

[8] Chen X., Wales P., Quinti L., Zuo F., Moniot S., Herisson F., Rauf N.A., Wang H., Silverman R.B., Ayata C., Maxwell M.M., Steegborn C., Schwarzschild M.A., Outeiro T.F., Kazantsev A.G. (2015). The sirtuin-2 inhibitor AK7 is neuroprotective in models of Parkinson’s disease but not amyotrophic lateral sclerosis and cerebral ischemia. PLoS One.

[9] Choong C.-J., Sasaki T., Hayakawa H., Yasuda T., Baba K., Hirata Y., Uesato S., Mochizuki H. (2016). A novel histone deacetylase 1 and 2 isoform-specific inhibitor alleviates experimental Parkinson's disease. Neurobiol. Aging.

[10] Chuang D.-M., Leng Y., Marinova Z., Kim H.-J., Chiu C.-T. (2009). Multiple roles of HDAC inhibition in neurodegenerative conditions. Trends Neurosci..

[11] Dexter D.T., Jenner P. (2013). Parkinson disease: from pathology to molecular disease mechanisms. Free Radic. Biol. Med..

[12] Di Fruscia P., Zacharioudakis E., Liu C., Moniot S., Laohasinnarong S., Khongkow M., Harrison I.F., Koltsida K., Reynolds C.R., Schmidtkunz K., Jung M., Chapman K.L., Steegborn C., Dexter D.T., Sternberg M.J.E., Lam E.W.F., Fuchter M.J. (2015). The discovery of a highly selective 5,6,7,8-tetrahydrobenzo[4,5]thieno[2,3-d]pyrimidin-4(3H)-one SIRT2 inhibitor that is neuroprotective in an in vitro Parkinson’s disease model. ChemMedChem.

[13] Dietz K.C., Casaccia P. (2010). HDAC inhibitors and neurodegeneration: at the edge between protection and damage. Pharmacol. Res..

[14] Donmez G., Outeiro T.F. (2013). SIRT1 and SIRT2: emerging targets in neurodegeneration. EMBO Mol. Med..

[15] Durrenberger P.F., Fernando F.S., Magliozzi R., Kashefi S.N., Bonnert T.P., Ferrer I., Seilhean D., Nait-Oumesmar B., Schmitt A., Gebicke-Haerter P.J., Falkai P., Grünblatt E., Palkovits M., Parchi P., Capellari S., Arzberger T., Kretzschmar H., Roncaroli F., Dexter D.T., Reynolds R. (2012). Selection of novel reference genes for use in the human central nervous system: a BrainNet Europe Study. Acta Neuropathol. (Berl.).

[16] Garske A.L., Smith B.C., Denu J.M. (2007). Linking SIRT2 to parkinson’s disease. ACS Chem. Biol..

[17] Guan Q., Wang M., Chen H., Yang L., Yan Z., Wang X. (2016). Aging-related 1-methyl-4-phenyl-1,2,3,6-tetrahydropyridine-induced neurochemial and behavioral deficits and redox dysfunction: improvement by AK-7. Exp. Gerontol..

[18] Gupta S.C., Sundaram C., Reuter S., Aggarwal B.B. (2010). Inhibiting NF-κB activation by small molecules as a therapeutic strategy. Biochimica et biophysicaacta.

[19] Hahnen E., Hauke J., Tränkle C., Eyüpoglu I.Y., Wirth B., Blümcke I. (2008). Histone deacetylase inhibitors: possible implications for neurodegenerative disorders. Expert Opin. Investig. Drugs.

[20] Harrison I.F., Anis H.K., Dexter D.T. (2016). Associated degeneration of ventral tegmental area dopaminergic neurons in the rat nigrostriatal lactacystin model of parkinsonism and their neuroprotection by valproate. Neurosci. Lett..

[21] Harrison I.F., Crum W.R., Vernon A.C., Dexter D.T. (2015). Neurorestoration induced by the HDAC inhibitor sodium valproate in the lactacystin model of Parkinson's is associated with histone acetylation and upregulation of neurotrophic factors. Br. J. Pharmacol..

[22] Harrison I.F., Dexter D.T. (2013). Epigenetic targeting of histone deacetylase: therapeutic potential in Parkinson's disease?. Pharmacol. Ther..

[23] Hirsch E.C., Breidert T., Rousselet E., Hunot S., Hartmann A., Michel P.P. (2003). The role of glial reaction and inflammation in parkinson's disease. Ann. N. Y. Acad. Sci..

[24] Hunot S., Brugg B., Ricard D., Michel P.P., Muriel M.-P., Ruberg M., Faucheux B.A., Agid Y., Hirsch E.C. (1997). Nuclear translocation of NF-κB is increased in dopaminergic neurons of patients with Parkinson disease. Proc. Natl. Acad. Sci. U. S. A..

[25] Inoue T., Hiratsuka M., Osaki M., Oshimura M. (2007). The molecular biology of mammalian SIRT proteins: SIRT2 functions on cell cycle regulation. ABBV Cell Cycle.

[26] Jenner P., Olanow C.W. (2006). The pathogenesis of cell death in Parkinson's disease. Neurology.

[27] Jian W., Wei X., Chen L., Wang Z., Sun Y., Zhu S., Lou H., Yan S., Li X., Zhou J., Zhang B. (2017). Inhibition of HDAC6 increases acetylation of peroxiredoxin1/2 and ameliorates 6-OHDA induced dopaminergic injury. Neurosci. Lett..

[28] Kannan V., Brouwer N., Hanisch U.-K., Regen T., Eggen B.J.L., Boddeke H.W.G.M. (2013). Histone deacetylase inhibitors suppress immune activation in primary mouse microglia. J. Neurosci. Res..

[29] Kazantsev A.G., Thompson L.M. (2008). Therapeutic application of histone deacetylase inhibitors for central nervous system disorders. Nat. Rev. Drug Discov..

[30] Kiernan R., Brès V., Ng R.W.M., Coudart M.-P., El Messaoudi S., Sardet C., Jin D.-Y., Emiliani S., Benkirane M. (2003). Post-activation turn-off of NF-κB-dependent transcription is regulated by acetylation of p65. J. Biol. Chem..

[31] Kontopoulos E., Parvin J.D., Feany M.B. (2006). α-synuclein acts in the nucleus to inhibit histone acetylation and promote neurotoxicity. Hum. Mol. Genet..

[32] Liu L., Arun A., Ellis L., Peritore C., Donmez G. (2012). Sirtuin 2 (SIRT2) enhances 1-methyl-4-phenyl-1,2,3,6-tetrahydropyridine (MPTP)-induced nigrostriatal damage via deacetylating forkhead box O3a (Foxo3a) and activating bim protein. J. Biol. Chem..

[33] Nakamura Y., Si Q.S., Kataoka K. (1999). Lipopolysaccharide-induced microglial activation in culture: temporal profiles of morphological change and release of cytokines and nitric oxide. Neurosci. Res..

[34] Outeiro T.F., Kontopoulos E., Altmann S.M., Kufareva I., Strathearn K.E., Amore A.M., Volk C.B., Maxwell M.M., Rochet J.-C., McLean P.J., Young A.B., Abagyan R., Feany M.B., Hyman B.T., Kazantsev A.G. (2007). Sirtuin 2 inhibitors rescue α-synuclein-mediated toxicity in models of Parkinson's disease. Science.

[35] Pais T.F., Szegő É.M., Marques O., Miller-Fleming L., Antas P., Guerreiro P., de Oliveira R.M., Kasapoglu B., Outeiro T.F. (2013). The NAD-dependent deacetylase sirtuin 2 is a suppressor of microglial activation and brain inflammation. EMBO J..

[36] Paiva I., Pinho R., Pavlou M.A., Hennion M., Wales P., Schütz A.-L., Rajput A., É. Szegő M., Kerimoglu C., Gerhardt E., Rego A.C., Fischer A., Bonn S., Outeiro T.F. (2017). Sodium butyrate rescues dopaminergic cells from alpha-synuclein-induced transcriptional deregulation and DNA damage. Hum. Mol. Genet..

[37] Peng G.-S., Li G., Tzeng N.-S., Chen P.-S., Chuang D.-M., Hsu Y.-D., Yang S., Hong J.-S. (2005). Valproate pretreatment protects dopaminergic neurons from LPS-induced neurotoxicity in rat primary midbrain cultures: role of microglia. Mol. Brain. Res..

[38] Pinho B.R., Reis S.D., Guedes-Dias P., Leitão-Rocha A., Quintas C., Valentão P., Andrade P.B., Santos M.M., Oliveira J.M.A. (2016). Pharmacological modulation of HDAC1 and HDAC6 in vivo in a zebrafish model: therapeutic implications for Parkinson’s disease. Pharmacol. Res..

[39] Qin H., Wilson C.A., Lee S.J., Zhao X., Benveniste E.N. (2005). LPS induces CD40 gene expression through the activation of NF-κB and STAT-1α in macrophages and microglia. Blood.

[40] Repetto G., d. Peso A., Zurita J.L. (2008). Neutral red uptake assay for the estimation of cell viability/cytotoxicity. Nat. Protoc..

[41] Righi M., Mori L., Libero G.D., Sironi M., Biondi A., Mantovani A., Donini S.D., Ricciardi-Castagnoli P. (1989). Monokine production by microglial cell clones. Eur. J. Immunol..

[42] Rothgiesser K.M., Erener S., Waibel S., Lüscher B., Hottiger M.O. (2010). SIRT2 regulates NF-κB-dependent gene expression through deacetylation of p65 Lys310. J. Cell Sci..

[43] Rouaux C., Jokic N., Mbebi C., Boutillier S., Loeffler J.-P., Boutillier A.-L. (2003). Critical loss of CBP/p300 histone acetylase activity by caspase-6 during neurodegeneration. EMBO J..

[44] Roy A., Ghosh A., Jana A., Liu X., Brahmachari S., Gendelman H.E., Pahan K. (2012). Sodium phenylbutyrate controls neuroinflammatory and antioxidant activities and protects dopaminergic neurons in mouse models of Parkinson’s disease. PLoS One.

[45] Saha R.N., Pahan K. (2006). HATs and HDACs in neurodegeneration: a tale of disconcerted acetylation homeostasis. Cell Death Differ..

[46] Scuderi C., Stecca C., Bronzuoli M.R., Rotili D., Valente S., Mai A., Steardo L. (2014). Sirtuin modulators control reactive gliosis in an in vitro model of Alzheimer’s disease. Front. Pharmacol..

[47] Sharma S., Taliyan R. (2015). Targeting histone deacetylases: a novel approach in Parkinson’s disease. Parkinson’s Disease.

[48] Spillantini M.G., Schmidt M.L., Lee V.M.-Y., Trojanowski J.Q., Jakes R., Goedert M. (1997). α − synuclein in lewy bodies. Nature.

[49] Suh H.S., Choi S., Khattar P., Choi N., Lee S.C. (2010). Histone deacetylase inhibitors suppress the expression of inflammatory and innate immune response genes in human microglia and astrocytes. J. Neuroimmune Pharmacol..

[50] Suo H., Wang P., Tong J., Cai L., Liu J., Huang D., Huang L., Wang Z., Huang Y., Xu J., Ma Y., Yu M., Fei J., Huang F. (2015). NRSF is an essential mediator for the neuroprotection of trichostatin A in the MPTP mouse model of Parkinson's disease. Neuropharmacology.

[51] Suuronen T., Huuskonen J., Pihlaja R., Kyrylenko S., Salminen A. (2003). Regulation of microglial inflammatory response by histone deacetylase inhibitors. J. Neurochem..

[52] Wang X., Guan Q., Wang M., Yang L., Bai J., Yan Z., Zhang Y., Liu Z. (2015). Aging-related rotenone-induced neurochemical and behavioral deficits: role of SIRT2 and redox imbalance, and neuroprotection by AK-7 Drug Design. Develop. Therapy.

[53] Wu J.Y., Niu F.N., Huang R., Xu Y. (2008). Enhancement of glutamate uptake in 1-methyl-4-phenylpyridinium-treated astrocytes by trichostatin A. Neuroreport.

[54] Wu X., Chen P.S., Dallas S., Wilson B., Block M.L., Wang C.-C., Kinyamu H., Lu N., Gao X., Leng Y., Chuang D.-M., Zhang W., Lu R.B., Hong J.-S. (2008). Histone deacetylase inhibitors up-regulate astrocyte GDNF and BDNF gene transcription and protect dopaminergic neurons. Int. J. Neuropsychopharmacol..

