# Supplementary material for: Pathological histone acetylation in Parkinson’s disease: Neuroprotection and inhibition of microglial activation through SIRT 2 inhibition
Source: Neurosci Lett. 2018 Feb 14;666:48–57. doi: 10.1016/j.neulet.2017.12.037 (PMC5821898; doi:10.1016/j.neulet.2017.12.037)
Supplement: Supplementary file 1 [file mmc1.docx]

**Supplementary Table 1 – Details of PD Patients and Control Individuals Who Donated Brain Tissue Utilised in this Study**

Means and SEMs are denoted in bold and italic respectively at the bottom of each column. Abbreviations: PMI, post-mortem delay; AAD, age at death; AAO, age at onset; DD, disease duration; ePD, early Parkinson’s disease; lPD, late Parkinson’s disease.

| **Control**  **Case** | | **Gender** | | **PMI (hrs)** | | | **AAD (yrs)** | | **Cause of Death** | | | | | | | | |  |  |
| --- | --- | --- | --- | --- | --- | --- | --- | --- | --- | --- | --- | --- | --- | --- | --- | --- | --- | --- | --- |
| **C01** | | ♀ | | 17 | | | 71 | | Myocardial infarction | | | | | | | | |  |  |
| **C02** | | ♀ | | 23 | | | 78 | | Unknown | | | | | | | | |  |  |
| **C03** | | ♀ | | 23 | | | 80 | | Breast carcinoma with spinal metastasis; carcinosarcoma uterus | | | | | | | | |  |  |
| **C04** | | ♀ | | 11 | | | 84 | | Pancreatic cancer | | | | | | | | |  |  |
| **C05** | | ♂ | | 17 | | | 77 | | Conductive cardiac failure. Chronic kidney disease. Osteoporosis. Malignant neoplasm of prostate | | | | | | | | |  |  |
| **C06** | | ♂ | | 12 | | | 90 | | Respiratory failure secondary to bronchial cancer | | | | | | | | |  |  |
| **C07** | | ♀ | | 28 | | | 80 | | End stage primary pulmonary hypertension | | | | | | | | |  |  |
| **C08** | | ♀ | | 22 | | | 89 | | Not reported | | | | | | | | |  |  |
| **C09** | | ♂ | | 22 | | | 88 | | Prostate cancer, bone metastases | | | | | | | | |  |  |
| **C10** | | ♂ | | 5 | | | 84 | | Bladder cancer, pneumonia | | | | | | | | |  |  |
| n=10 | | **4**♂**:6**♀ | | **18**  *2.2* | | | **82.1**  *1.9* | |  | | | | | | | | |  |  |
| **ePD**  **Case** | | **Gender** | | | **PMI (hrs)** | | **AAD (yrs)** | | **AAO (yrs)** | | | **DD (yrs)** | | **Braak (stage)** | | **Cause of Death** | | | **PD Medication** |
| **ePD01** | | ♂ | | | 22 | | 78 | | 70 | | | 9 | | 3 | | Pneumonia | | | Sinemet, Orphenadrine, Amantadine |
| **ePD02** | | ♂ | | | 21 | | 79 | | 67 | | | 12 | | 3 | | Not reported | | | Ropinirole, Madopar, Artane, Amitriptyline, Hyoscine, Risperidone |
| **ePD03** | | ♀ | | | 14 | | 76 | | 65 | | | 12 | | 4 | | Not reported | | | Selegiline, Madopar, Cabergoline |
| **ePD04** | | ♂ | | | 10 | | 76 | | 66 | | | 10 | | 3 | | Unknown | | | Madopar |
| **ePD05** | | ♀ | | | 10 | | 80 | | 67 | | | 13 | | 4 | | Not reported | | | Sinemet, Entacapone, Sinemet, Selegiline |
| **ePD06** | | ♀ | | | 22 | | 87 | | 77 | | | 9 | | 4 | | Gastrointestinal bleeding | | | Madopar, Amantadine, Selegiline |
| **ePD07** | | ♂ | | | 9 | | 72 | | 66 | | | 6 | | 4 | | Cardio-respiratory arrest; aspiration pneumonia | | | Pergolide, Selegiline, Sinemet , Sinemet, Cabergoline |
| **ePD08** | | ♀ | | | 5 | | 86 | | 68 | | | 18 | | 3 | | Not reported | | | Selegiline, Madopar, Cabergoline, Roprinirole |
| n=8 | | **4**♂**:4**♀ | | | **14.1**  *2.4* | | **79.3**  *1.8* | | **68.3**  *1.4* | | | **11.5**  *1.3* | | **3.5**  *0.2* | |  | | |  |
| **lPD**  **Case** | | **Gender** | | **PMI (hrs)** | | | **AAD (yrs)** | | | **AAO (yrs)** | **DD (yrs)** | | **Braak (stage)** | | **Cause of Death** | | | **PD Medication** | |
| **lPD01** | | ♀ | |  | | | 87 | | | 76 | 12 | | 6 | | Not reported | | | Madopar, Sinemet, Selegiline | |
| **lPD02** | | ♀ | | 14 | | | 85 | | | 67 | 18 | | 6 | | Bronchopneumonia and breast cancer with metastasis | | | Madopar, Sinemet, Bromocriptine, Pergolide, Lysuride, Selegiline | |
| **lPD03** | | ♂ | | 28 | | | 82 | | | 75 | 7 | | 6 | | Not reported | | | Sinemet, Pramipexole | |
| **lPD04** | | ♂ | | 14 | | | 82 | | | 65 | 18 | | 6 | | Not reported | | | Sinemet preparations, Cabergoline, Bromocriptine, Pergolide, Selegiline | |
| **lPD05** | | ♂ | | 6 | | | 77 | | | 67 | 10 | | 6 | | Not reported | | | Selegiline; Benzhexol; Sinemet; Pergolide; Pramipexole; Quetiapine; Paroxetine. | |
| **lPD06** | | ♂ | | 16 | | | 80 | | | 60 | 19 | | 6 | | Not reported | | | Sinemet, Ropinirole, Selegiline, Entacapone, Tolcapone, Cabergoline | |
| **lPD07** | | ♂ | | 10 | | | 83 | | | 74 | 9 | | 6 | | Not reported | | | Madopar Sinemet, Selegiline, Pergolide, Ropinirole, Tolcapone, Entacapone | |
| **lPD08** | | ♀ | | 22 | | | 78 | | | 59 | 19 | | 6 | | Chest infection | | | Fludrocortisone, Sulpiride, Sinemet, Dothiepin, Selegiline, Madopar, Dispersible, Amitriptyline, Madopar, Sertraline, Artane, Propranolol | |
| **lPD09** | | ♀ | | 22 | | | 81 | | | 67 | 14 | | 6 | | Not reported | | | Madopar, Madopar, Cabergoline, Sulpiride, Selegiline, Olanzapine, Amantadine | |
| **lPD10** | | ♂ | | 10 | | | 82 | | | 72 | 11 | | 6 | | Pneumonia, fractured neck of femur, pulmonary embolisms, COPD | | | Pramipexole, Benzhexol, Sinemet, Madopar | |
| **lPD11** | | ♂ | | 15 | | | 75 | | | 50 | 25 | | 6 | | Pancreatic cancer | | | Sinemet, Madopar Dispersible, Madopar, Madopar, Pergolide, Entacapone, Disipal, Galantamine, Benzhexol, Bromocriptine, Selegiline | |
| **lPD12** | | ♀ | | 15 | | | 85 | | | 70 | 15 | | 6 | | Not reported | | | Sinemet, Selegiline, Risperidone, Pergolide, Haloperidol, Rivastigmine | |
| n=12 | | **7**♂**:5**♀ | | **15.6**  *1.9* | | | **81.4**  *1.0* | | | **66.8**  *2.2* | **14.8**  *1.5* | | **6**  *0* | |  | | |  | |

**Supplementary Table 2 – Probe and Primer Sequences of** **PrimeTime™ qPCR Assays**

All PrimeTime™ qPCR Assays were obtained from Integrated DNA Technologies (USA) and contained 2.5nM of probe, 5nm of primer 1 and 5nM of primer 2. Abbreviations: A, adenosine; C, cytosine; G, guanine; T, thymine; HEX™, Hexachlorofluorescein; IABkFQ, Iowa Black® FQ; Fwd, forward; Rev, reverse.

| Gene Name | Protein Product | Sequence | |
| --- | --- | --- | --- |
| *XPNPEP1* | XPNPEP1 | Probe | **5'-/5HEX/CTTTGGGAA/ZEN/CCTCTTTCTCCAGCCA/3IABkFQ/-3'** |
|  |  | Fwd Primer | *5'-CGAAACTCCTCAGCTTTGTCA-3'* |
|  |  | Rev Primer | *5'-CTGTTGCTCTCTGTGAACTCT-3'* |
| *TH* | TH | Probe | **5'-/56-FAM/ACGTCTCAA/ZEN/ACACCTTCACAGCTCG/3IABkFQ/-3'** |
|  |  | Fwd Primer | *5'-GGTCTCTAGATGGTGGATTTTGG -3'* |
|  |  | Rev Primer | *5'-TGCTAAACCTGCTTCTTCTCC -3'* |
| *HLADPA1* | HLA-DPα1 | Probe | **5'-/56-FAM/CCTAAGTCC/ZEN/TCTTCTGTTCAGATATTTTGTCACC/3IABkFQ/-3'** |
|  |  | Fwd Primer | *5'-GTTTGTAGGGCAGCTGGAG-3'* |
|  |  | Rev Primer | *5'-CACCGTCCTCATCATAAAGTCTC -3'* |
| *SIRT2* | Sirtuin2 | Probe | **5'-/56-FAM/TGACCTTGG/ZEN/AAGGGGTGGCC/3IABkFQ/-3'** |
|  |  | Fwd Primer | *5'-CTCCCACCAAACAGATGACTC -3'* |
|  |  | Rev Primer | *5'-TCTCCCAGACGCTCA -3'* |

**Supplementary Figure 1 – Characterisation of Cell Lines**

(A) TH and NeuN protein content of lysates from N27 cells seeded at increasing densities were quantified for confirmation of N27 expression of markers of dopaminergic neurons. (B) Iba-1 protein content of lysates from N9 cells seeded at increasing densities were quantified using Western blot analysis for confirmation of N9 expression of a marker for microglial cells.

**
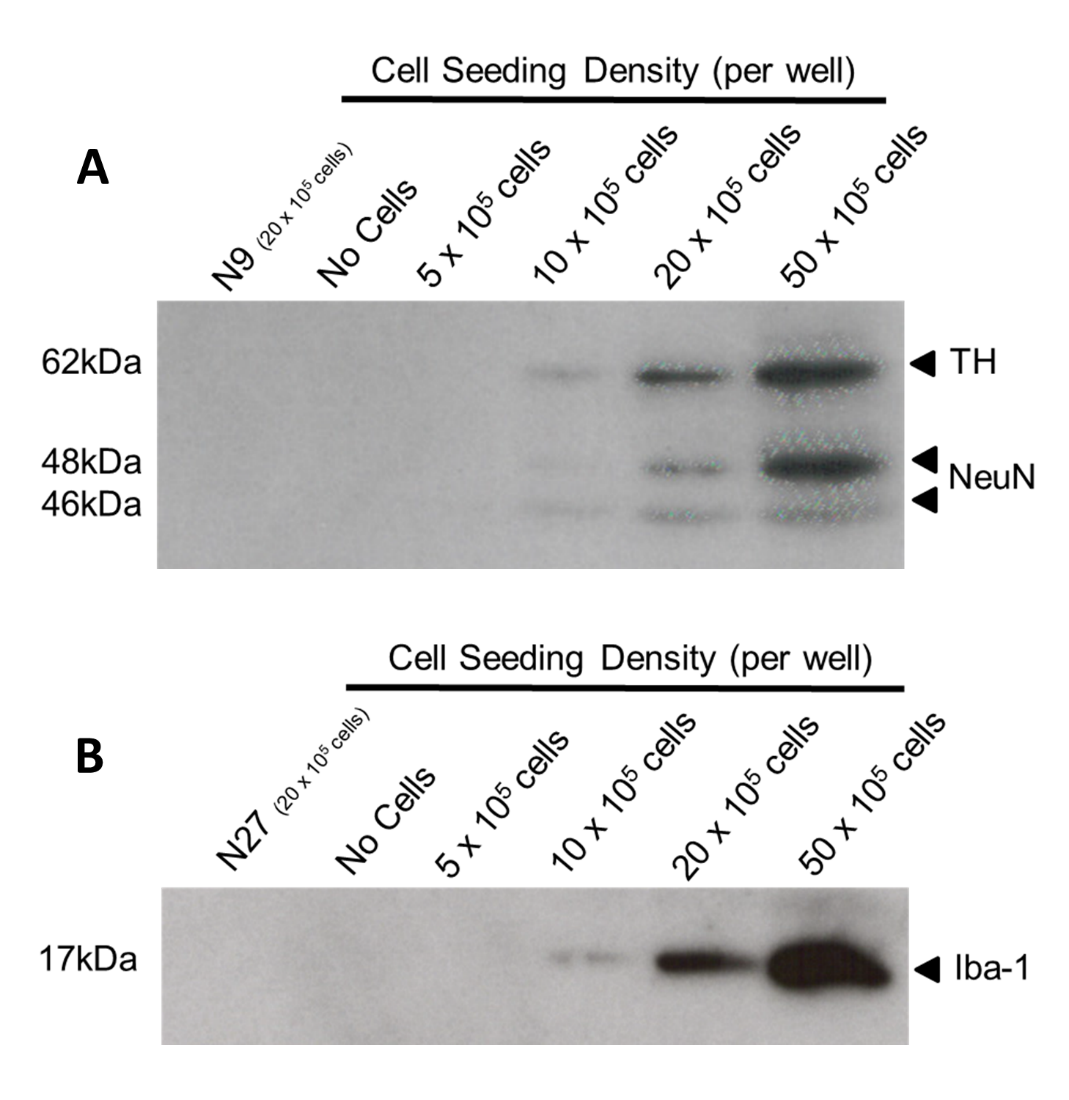
**

**Supplementary Figure 2 – AGK2 Treatment Optimisation**

AcH3-Lys9 content of lysates from cells treated with AGK2 (1μM) for a range of incubation periods were quantified by Western blot analysis. Densitometry analysis of AcH3-Lys9 bands from (a) N27 cells and (B) N9 cells, relative to β-actin used as a loading control demonstrate that histone acetylation increases with incubation time with AGK2. Crossed bars indicate chosen time periods for subsequent experiments. (C) Representative blot of data presented in (A). (D) Representative blot of data presented in (B). N=3 independent replicates. Statistical significance indicated with asterisks: *p<0.05, **p<0.01.

**
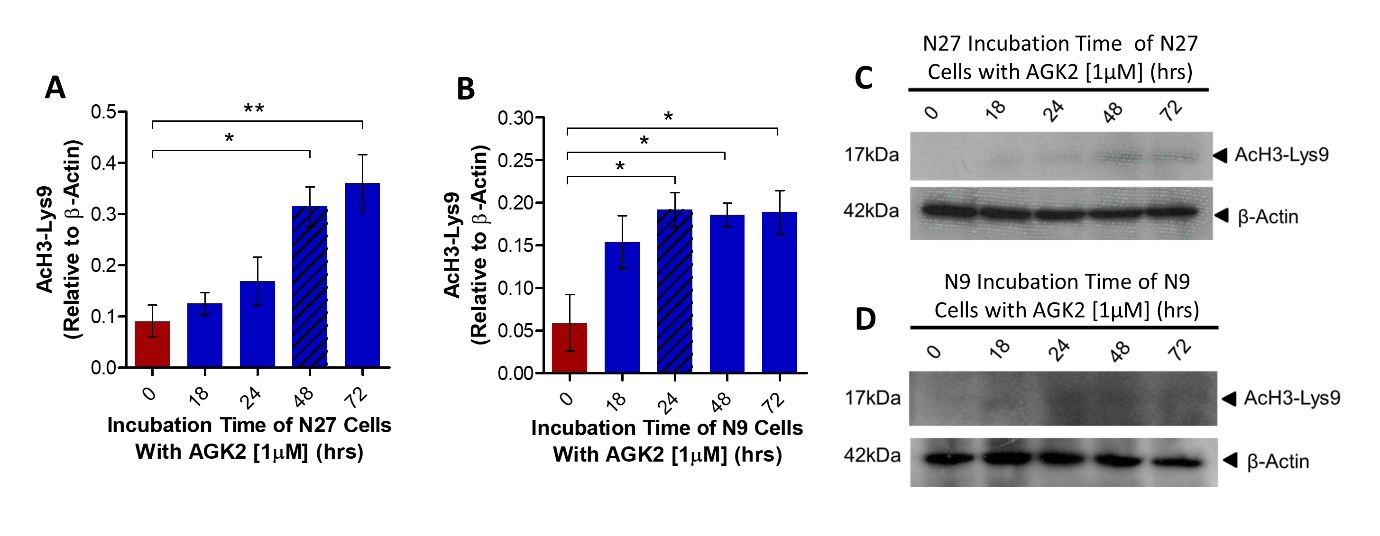
**
